# The ameliorative effect of monotropein, astragalin, and spiraeoside on oxidative stress, endoplasmic reticulum stress, and mitochondrial signaling pathway in varicocelized rats

**DOI:** 10.1186/s12906-019-2736-9

**Published:** 2019-11-26

**Authors:** Keshab Kumar Karna, Bo Ram Choi, Jae Hyung You, Yu Seob Shin, Wan Shou Cui, Sung Won Lee, Ji Hoon Kim, Chul Young Kim, Hye Kyung Kim, Jong Kwan Park

**Affiliations:** 1 Department of Urology, Institute for Medical Sciences, Chonbuk National University Medical School-Biomedical Research Institute and Clinical Trial Center for Medical Device, Chonbuk National University Hospital, Jeonju, 54907 Republic of Korea; 2 Andrology Center, Peking University First Hospital, Beijing, People’s Republic of China; 3 Department of Urology, Samsung Medical Center, Samsung Biomedical Research Institute, Sungkyunkwan University School of Medicine, Seoul, 06351 Republic of Korea; 4 College of Pharmacy, Hanyang University, Ansan, 426-791 Republic of Korea; 5 College of Pharmacy, Kyungsung University, Busan, 48434 Republic of Korea

**Keywords:** Varicocele, MAS, Oxidative stress, ER stress, Mitochondria, Apoptosis

## Abstract

**Background:**

Monotropein, astragalin, and spiraeoside (MAS) are active compounds extracted from medicinal herbs; monotropein from Morinda officinalis How (Rubiaceae), astragalin (kaempferol 3-O-glucoside) from Cuscuta chinensis Lamark (Convolvulaceae) and spiraeoside from the outer scales of *Allium cepa* L. (Liliceae) in a ratio of 6.69:0.41:3.61. Monotropein, astragalin, and spiraeoside are well-known antioxidants, anti-inflammatory, and antinociceptive agents. The current investigation aims to study the molecular mechanism of varicocele-induced male infertility and the underlying pharmacological mechanisms of MAS.

**Methods:**

Four groups were included: control (CTR), MAS 200 group (MAS 200 mg/kg), varicocele group (VC), and VC + MAS 200 group (MAS 200 mg/kg). Sprague-Dawley (SD) rats were treated with 200 mg/kg MAS or vehicle once daily for 28 days. The possible signaling mechanism and effects of MAS were measured via histological staining, immunohistochemistry, western blot, and biochemical assays.

**Results:**

Parameters such as sperm motility and count, Johnsen’s scores, spermatogenic cell density, serum testosterone, testicular superoxide dismutase (SOD), catalase, glutathione peroxidase (GPx) and expression of the steroidogenic acute regulatory protein (StAR) improved significantly in the VC + MAS 200 group compared with the VC group. MAS treatment of varicocele-induced group significantly decreased the levels of serum luteinizing hormone (LH) and follicle-stimulating hormone (FSH), as well as testicular interleukin-6 (IL6), tumor necrosis factor-α (TNF-α), ROS/RNS, and malondialdehyde (MDA). It also decreased the apoptotic index and reduced the expression of endoplasmic reticulum (ER) protein levels (Grp78, p-IRE1α, and p-JNK) and apoptotic markers such as cleaved caspase-3 and Bax/Bcl2 ratio.

**Conclusion:**

This study suggests that the crosstalk between oxidative stress, ER stress, and mitochondrial pathway mediates varicocele-induced testicular germ cell apoptosis. MAS promotes spermatogenesis in varicocele-induced SD rat, probably by decreasing cytokines (IL-6, TNF-α) levels, regulating abnormal sex hormones, and decreasing oxidative stress, ER stress, and apoptosis.

## Background

A varicocele (VC) is defined by the abnormal dilation of internal testicular vein and pampiniform venous plexus within the spermatic cord with blood reflux [1]. VC is the common cause of male infertility and is prevalent in approximately 15 to 20% of male population, including 35% of males with primary infertility and 70–80% of males with secondary infertility [2–4]. Clinically detectable VC is associated with progressive testicular damage and infertility. VC is the leading cause of abnormal semen, low sperm count, decreased sperm motility and abnormal sperm morphology [5]. It is also associated with testicle shrinkage and pain [6]. About 2 to 10% of males with varicocele suffer from scrotal pain [6]. VC–induced impairment of testicular function is associated with pathophysiological events such as testicular hyperthermia, testicular hypoxia, oxidative stress, endoplasmic reticulum (ER) stress and apoptosis [7–9]. The relationship between VC and infertility is still unclear at the molecular level. However, many studies explained the incidence of varicocele is based on multiple mechanisms.

VC-induced testicular apoptosis has been recognized as a cause of male infertility [10]. ER stress signaling plays a crucial role in cell survival and apoptosis. The ER stress signaling pathway is activated by multiple pathophysiological conditions and toxic insults [11, 12]. It is well known that oxidative stress plays a key role in VC-induced testicular dysfunction [13]. Oxidative stress or reactive oxygen species (ROS) triggers ER stress signaling and ER stress in turn exacerbates ROS production [14]. Prolonged ER stress triggers apoptosis via activation of C/EBP homologous protein (CHOP), c-Jun amino terminal kinase (JNK) and caspase-12 pathway in humans and cleaved caspase-12 in mice [15]. The intrinsic or mitochondrial pathway is also linked to cellular stress and apoptosis in varicocele-induced rat testis [16].

Currently, many studies suggest that natural extracts with antioxidant properties may alleviate VC-induced male infertility in rat models [17, 18]. MAS is a mixture of three chemical compounds (monotropein, astragalin and spiraeoside) isolated form MOTILIPERM, [12, 19] a mixture of herbal plant extracts. MOTILIPERM is obtained from three plant extracts- *roots of Morinda officinalis* How (Rubiaceae), outer scales of *Allium cepa* L. (Liliaceae) and seeds of *Cuscuta chinensis* Lamark (convolvulaceae) [12]. Major marker components of herbal ingredients in MOTILIPERM include monotropein, diacetyl asperulosidic acid, hyperoside, kaempferol 3-*O*-glucoside, quercetin 4′-*O*-glucoside and quercetin [8]. Monotropein is a major iridoid glycoside derived from the roots of *Morinda officinalis* How (Rubiaceae) and exhibits anti-inflammatory and antinociceptive activity [20]. Treatment of *M. officinalis* polysaccharides promotes spermatogenesis in experimental VC rats by regulating hypothalamic gonadotropin-releasing hormone (GnRH), repairing the damage of tight junction protein expression and decreasing inflammatory cytokines levels [21, 22]. Another study reported that *M. officinalis* polysaccharides attenuate VC-induced testicular dysfunction by modulating angiogenesis [23]. Astragalin (3-*O*-glucoside of kaempferol) is a flavonoid isolated from the seeds of *Cuscuta chinensis* Lamark (convolvulaceae) and has been used to improve male reproductive function [12]. Seeds of *Cuscuta chinensis* exhibits antinociceptive and anti-inflammatory activities [24]. Previous study has demonstrated astragalin as the active compounds in seeds of *Cuscuta chinensis* [8, 12, 25]. Spiraeoside (quercetin, quercetin 3.4′-diglucoside) is the predominant flavonoid present in the outer scales of *Allium cepa* L. (Liliaceae) and prevents oxidation of low density lipoproteins by scavenging the free oxygen radicals [26, 27]. Treatment of scale of *Allium cepa* L. (Liliaceae) alleviates spermatogenesis in experimental VC and adriamycin-induced testicular toxicity by regulating oxidative stress [8, 28]. The protective effect of MAS against varicocele-induced testicular dysfunction has never been investigated.

A study from our lab reported the beneficial effect of MOTILIPERM against varicocele-induced testicular toxicity [8]. In the present study, the mixture of three major compounds- monotropein, astragalin and spiraeoside (MAS) were derived from MOTILIPERM, and its efficacy in improving fertility was investigated. The aim of the present study was to investigate the underling mechanism related to oxidative stress, ER stress and mitochondrial apoptosis in varicocele-induced SD rats and to determine the protective effect of MAS against infertility.

## Methods

### Animals and experimental protocol

The Animal Care and Ethics Committee of Chonbuk National University Laboratory Animal Center, accredited by the Association for Assessment and Accreditation of Laboratory Animal Care (AAALAC) (cuh-IACUC-2017-13) approved all the experiments. A total of 40 sexually mature male Sprague-Dawley rats weighing from 210 to 240 g were supplied by KOATECH, Gyeonggi-do, South Korea. The animals were fed a standard rat chow diet and had free access to water ad libitum. Rats were maintained in the animal facility under standard living conditions (20 ± 2 °C, relative humidity of 50 ± 10% with a 12-h light/dark photoperiod). Rats were acclimated to the laboratory environment for the first week.

After one week of acclimatization, male SD rats were randomly divided into four groups (10 rats per group): 1) control (CTR) group, 2) MAS 200 mg/kg p.o. (MAS 200) group, 3) varicocele (VC) group, and 4) VC + MAS 200 mg/kg p.o. (VC + MAS 200) group. Varicocele was induced in the left testis of rats in the VC and VC + MAS 200 groups as previously described [8]. The CTR and MAS 200 groups underwent midline abdominal incision without ligation of left renal vein, or the left internal spermatic vein (ISV) to common iliac vein and communicating branches of left ISV. MAS was administered after 4 weeks of varicocele induction. MAS 200 was dissolved in sterile normal saline and oral administration by gavage needle **(**JD-S-124, Jeungdo, Seoul, Korea) at 200 mg/kg per day in MAS 200 and VC + MAS 200 groups. The CTR and VC groups received normal saline (vehicle) for 28 days. All male rats were anesthetized 48 h prior to the last treatment. Rats were anesthetized by ketamine (100 mg/mL) and 2% xylazine hydrochloride (20 mg/mL). Blood samples were collected from the rats’ vena cava. Serum was prepared and stored at -80 °C for further biochemical analysis. Body weight and reproductive organ weight were measured. The testicular tissue, epididymis, seminal vesicle, prostate and penis were collected and placed in Bouin’s solution and liquid nitrogen for further analysis. At the end of the experiment rats were killed by hypothermia and cervical dislocation.

### Chemicals and reagents

All other chemical reagents were of analytical grade and obtained from standard commercial suppliers or as indicated in the specified methods.

### Preparation of MAS 200

MAS is a mixture of three pure compounds extracted from MOTILIPERM. It was prepared by mixing the three medicinal herb extracts from the roots of *Morinda officinalis* How (Rubiaceae), the seeds of *Cuscuta chinensis* Lamark (Convolvulaceae) and the outer scales of *Allium cepa* Linnaeus (Liliaceae). These three medicinal herbs were purchased from the Kyungdong Oriental herbal market, Seoul, Korea, in November 2016, and identified by one of the author (Chul Young Kim). A voucher specimen were deposited at the Herbarium of the College of Pharmacy, Hanyang University, South Korea with a name of Cinthera-1, Cinthera-2, and Cinthera-3, bearing voucher number HYUP-MO-001, HYUP-CC-001, and HYUP-AC-001, respectively. In our previous studies, MOTILIPERM doses of 200 mg/kg were reported to increase the sperm count and motility in rat models of cisplatin, finasteride, adriamycin and varicocele-induced infertility [8, 12, 19, 28]. The content of monotropein, astragalin and spiraeoside in 1 g doses of MOTILIPERM were 6.69 ± 0.19 mg/g, 0.41 ± 0.02 mg/g and 3.61 ± 0.08 mg/g, respectively (Additional file 5: Table S3). Thus, the MAS ratio was calculated according to 200 mg/kg of MOTILIPERM.

### Identification of major ingredients 1–7 in MOTILIPERM

Major ingredients of each herb were identified by high performance liquid chromatography-photodiode array-electrospray ionization mass spectrometry (HPLC-PDA/ESI-MS) respectively. The retention time, UV spectra, and mass spectrometry of major peaks were compared with those of MOTILIPERM (Additional files 1 and 2: Figures S1 and S2). A Waters 2695 Alliance HPLC system (Waters Corporation, Milford, MA, USA) equipped with a vacuum degasser, a binary pump, an autosampler, and a model 2996 photodiode array detector were used. The mass spectrometric identification was completed using a Micromass ZQ mass spectrometer (Manchester, UK). Chromatographic separation was accomplished on an Atlantis®RdC18 (2.1 mm × 150 mm, 3 μm,) analytical column. Water (A) and acetonitrile (B) were used as the mobile phase with the gradient elution mode as follows: 0–10 min, 0% B; 10–40 min, 0–30% B; 40–50 min, 30–50% B; 50–60 min, and 50–100% % B. The flow rate was set at 0.2 mL/min. The injection volume was 10 μl. UV spectra recorded were in the range of 210–400 nm. The micromass ZQ mass spectrometer equipped with an electrospray ionization source (ESI) probe working at 105 °C was operated in the positive or negative ion mode. Nitrogen was used as the desolvation gas at a flow rate of 250 Lh − 1. The desolvation temperature was 200 °C. Mass values of 200–1100 u were measured. Capillary and cone voltages were 3000 and 60 V for ESI+, and 3000 and 60 V for ESI−, respectively. Data acquisition and processing were performed using MassLynx 4.1.

### Quantification of active ingredients in MOTILIPERM

Three representative ingredients including monotropein (**1**), astragalin (**5**) and spiraeoside (**6**) from MOTILIPERM were purified by repeated chromatography and preparative HPLC (Additional file 1: Figure S1) and their chemical structure was elucidated by NMR and mass spectrometry data compared with previously reported data. The Agilent 1260 HPLC system with Shiseido C18 Capcell Pak UG 120 (250 mm × 4.6 mm, 5 μm, Shiseido, Tokyo, Japan) was used at a flow rate 1 mL min^− 1^ of mobile phase controlled by binary pumps at 40 °C. Water containing 0.1% trifluoroacetic acid (A) and acetonitrile containing 0.1% trifluoroacetic acid (B) were used as mobile phases in the gradient elution mode. The gradient elution was as follows: 0–10 min, 0% B; 10–40 min, 0–30% B; 40–50 min, 30–50% B; 50–60 min, 50–100% B. The effluent was monitored at 254 nm. Methanol stock solutions of three compounds were prepared and diluted to appropriate concentration for the construction of calibration curves. Linear regression analysis, limit of detection (LOD), limit of quantification (LOQ), intra and inter-day variability of monotropein, astragalin and spiraeoside in MOTILIPERM are presented in Additional files 3 and 4: Tables S1 and S2.

### Sperm count and sperm motility in the vas deferens and epididymis

Epididymis distal cauda and the vas deferens were excised, freed from the fat pad, blood vessels and connective tissue. The tissues was minced in separate 1.5 mL microcentrifuge tubes with pre-warm normal saline at 37 °C and incubate for 5 min to allow the dispersion of spermatozoa. Motile spermatozoa numbers within 10 squares of the grid were counted on a pre-warmed counting chamber under a light microscope at 20x magnification and the mean sperm count was recorded as millions of sperm per mL. Sperm motility (%) was evaluated within 3 to 5 min of placing a sperm suspension in a pre-warmed counting chamber under a light microscope at 20x magnification (SEFI-Medical Instruments, Haifa, Israel). The percentage of motile spermatozoa was assessed as previously described [19].

### Hormone levels, hematology and serum biochemistry

The concentrations of luteinizing hormone (LH), follicle-stimulating hormone (FSH) and serum testosterone were determined using the commercial enzyme-linked immunosorbent assay (ELISA) kits (E-EL-R0026, rat LH Elisa kit; E-EL-R0391, rat FSH Elisa kit; Elabscience, Houston, Texas, USA; 55-TESMS-E01, mouse/rat testosterone kit, ALOCO, 26-G Keewaydin Drive, Salem, NH, USA; E-EL-R0026) according to the manufacturers’ instructions. The complete blood counts (CBC) in anticoagulated blood samples were analyzed (Vet ABC, Heska, Loveland, CO). The activity of serum aspartate aminotransferase (AST) and alanine aminotransferase (ALT) was analyzed according to the International Federation of Clinical Chemistry reference method (ASAN Pharmaceutical Co., Ltd., Seoul, Korea).

### Testicular histopathology

Hematoxylin and eosin (H&E) staining was performed as described in our previous study [8]. Left testicular tissue was analyzed with standard light microscopy. Spermatogenic cell density was analyzed by measuring the thickness of the germinal cell layer and the diameter of the seminiferous tubules. The seminiferous tubules of H&E-stained sections were graded by Johnsen’s score under X400 optical microscope as previously described [19]. The damaged tubules at the edges of the section were excluded. Cross sections of a minimum of 30 seminiferous tubules from these slides were assessed for the presence of spermatogenic cells and assigned a score ranging from 1 to 10.

Apoptotic activities within the seminiferous tubules were estimated using the TUNEL assay (Dead End™ Colorimetric TUNEL System for qualitative study; G7132, Promega, Madison, WI, USA). The experiments were carried out as per manufacturer’s instruction. Two slides derived from each animal were used for quantitative analysis. In cross section, a minimum of 30 seminiferous tubules from each slide were counted to determine the number of apoptotic cells under an optical microscope (X40 objective). The apoptotic index (AI) was calculated as the percent of positive nuclei staining dark-brown under a light microscope.

### Immunohistochemistry

Serial sections of paraffin-embedded testis were deparaffinized, and subjected to 1X Target Retrieval Solution, pH 6.0 (DAKO, S1699, Glostrup, Denmark). Sections were incubated with peroxidase-blocking solution (DAKO, S2023) for 15 min at room temperature (RT) and washed with 1X PBS buffer for 5 min twice. Tissue sections were incubated with an anti-Grp78 and StAR protein antibody (rabbit monoclonal, 1: 100; Abcam Cambridge, MA USA, Anti-Grp78, catalog number: ab21685; Cell Signaling, Beverly, MA, USA; StAR, catalog number: D10H12;) for 24 h at 4 °C after blocking with serum block solution for 10 min at room temperature (DAKO, X0909). The slides were further rinsed in 1 X PBS and incubated with a secondary antibody (Anti-rabbit IgG; vector Labs, Burlingame, CA, USA; catalog number: MP-7451) for 1 h followed by AEC substrate chromogens (ImmPACT AEC Peroxidase substrate; vector Labs, Burlingame, CA, USA; catalog number: SK-4205) and washed with deionized water, and counter-stained with hematoxylin. The slides were washed with tap water and finally mounted with an aqueous medium (Abcam Cambridge, MA USA).

### Malondialdehyde level

The malondialdehyde (MDA) levels in testis tissue homogenates were determined using a MDA assay kit (NWLSSTM Malondialdehyde Assay kit; NWK-MDA01, Northwest Life Science Specialties LLC., Vancouver, WA, USA) according to the manufacturer’s instructions. Absorbance of the colored complex was measured via kinetic spectrophotometric analysis at optical density 532 nm. The MDA concentration level was analyzed by comparing the analyzed absorbance value to an MDA standard curve. The level of MDA was expressed as μmoles per mg tissue.

### Reactive oxygen species (ROS)/reactive nitrogen species (RNS) level

The ROS/RNS content in testis tissue homogenates was analyzed using commercially available fluorescence kit (STA-347, OxiSelect™ in vitro ROS/RNS assay kit, Cell Biolabs, Inc., San Diego, CA, USA). Absorbance of the reaction was measured using a SpectraMax Gemini XS Fluorimeter at excitation and emission wavelengths of 480 and 530 nm,. The experiments were carried out as per manufacturer instructions.

### Determining of antioxidant enzymes

Testicular tissues (100 mg) were washed with 1X PBS (pH 7.4) to remove excess blood thoroughly. The activity of SOD, GPx, and catalase in whole tissue supernatant was evaluated using commercially available kits (Cayman Chemical, Ann Arbor, MI, USA; item no.706002, superoxide dismutase kit; item no. 703102, glutathione peroxidase kit; item no. 707002, catalase assay kit) as per manufacturer’s instructions. Values were expressed as milligrams of protein.

### Cytokine measurements

Testicular tissues (100 mg) were washed with 1X PBS (pH 7.4) to remove excess blood thoroughly. Tissues were homogenized in 1 mL of 1X PBS on ice and stored at − 20 °C overnight. Two freeze-thaw cycles were then performed and the homogenate was centrifuged at 10,000 g for 15 min at 4 °C. The supernatant was used for assays. Concentrations of interleukin-6 (IL-6) and tumor necrosis factor-α (TNF-α) were measured by enzymatic methods using commercial kits as per manufacturer’s instructions (BMS625 IL-6 rat Elisa kit, BMS 622 rat TNF-α kit, Thermo Fisher Scientific, Waltham, MA, USA). Values were expressed in milligrams of protein.

### Western blotting

Protein isolation and western blot were conducted as described in our previous study [19]. Levels of ER stress markers [glucose-regulated protein-78 (GRP-78), phosphorylated c-Jun-N-terminal kinase (p-JNK), phosphorylated inositol-requiring transmembrane kinase/endoribonuclease 1α (p-IRE1α)], apoptosis markers [pro-caspase-3, cleaved caspase 3, B-cell lymphoma 2 (Bcl-2), and BCL 2 associated X protein (Bax)] and steroidogenic acute regulatory protein (StAR) were measured in the testicular tissue. A total of 30–60 μg of protein were loaded in each sample per lane, subjected to 8 to 12% SDS–polyacrylamide gel electrophoresis, followed by transferred onto PVDF membranes using trans-blot® SD semi-dry electrophoretic transfer cell (Bio-Rad, Hercules, CA, USA). Protein transferred membrane was then blocked with 5% bovine serum albumin (BSA) for an hour at room temperature and incubated overnight at 4 °C with the following primary antibodies: phosphorylated antibodies p-IRE1α (ab 48,187, Abcam Cambridge, MA USA) and p-JNK (SC-6254, Santa Cruz Biotechnology, Dallas, TX, USA), non-phosphorylated antibodies GRP-78 (ab 21,685, Abcam Cambridge, MA USA), pro-caspase-3, cleaved caspase 3, Bax, Bcl-2, StAR (catalog numbers: 9662S; 9664; 2772S; 3498; D10H12, Cell Signaling Technology, Beverly, MA, USA) in the presence of 5% non-fat milk. The membrane was washed with Tris-buffered saline containing 0.05% Tween 20 (TBST, pH 7.2) ten min each for three times prior to incubation with 1:5000 diluted secondary antibodies [anti-mouse, anti-rabbit (Cell Signaling Technology, Beverly, MA, USA) at room temperature for 1 h. The membrane was washed ten min each for three times with TBST. Antigen-antibody complexes were then visualized by ECL system (Vilber Lourmat, France).

### Statistical analyses

All data were expressed as the mean ± standard error of the mean (SEM) and were analyzed by one-way analysis of variance (ANOVA) followed by Tukey’s post hoc test (SPSS version 22; IBM, Armonk, NY, USA). *P* < 0.05 was considered statistically significant. GraphPad PRISM (Version 6, GraphPad Software, San Diego, CA, USA) was used for the graph analysis.

## Results

### Body and organ weight

The results of body and organ weights are presented in Table 1. There were no significant effects of body and organ weight among all the groups except for a significant decrease in the testicular weight in VC group compared with CTR group (*P <* 0.05).

**Table 1:** Effect of MAS 200 on body weight and reproductive organ weight in varicocele-induced male SD rats

Parameter	CTR	MAS 200	VC	VC + MAS 200
Body weight (sacrifice; g)	411.20 ± 5.14	427.20 ± 5.48	413.80 ± 3.23	432.70 ± 7.70
Testis weight (g)	2.09 ± 0.04	2.02 ± 0.05	1.79 ± 0.11*	2.01 ± 0.04
Epididymis weight (g)	0.70 ± 0.03	0.75 ± 0.01	0.68 ± 0.02	0.75 ± 0.02
Seminal vesicles weight (g)	1.33 ± 0.04	1.31 ± 0.05	1.20 ± 0.04	1.25 ± 0.04
Prostate weight (g)	0.99 ± 0.03	1.23 ± 0.05	1.12 ± 0.07	1.12 ± 0.07
Penis weight (g)	0.34 ± 0.01	0.33 ± 0.01	0.33 ± 0.01	0.32 ± 0.01
Kidney weight (g)	1.30 ± 0.01	1.35 ± 0.03	1.31 ± 0.02	1.33 ± 0.02

Data are presented in mean ± SEM. The differences were tested by one-way ANOVA followed by Tukey’s post hoc test; *n* = 10 for each group. ^*^Significant at *P* < 0.05 versus CTR group. *CTR* control, *MAS 200* MAS 200 mg/kg p.o., *VC* varicocele, *VC + MAS 200* MAS 200 mg/kg p.o., *p.o.* per oral, *ANOVA* analysis of variance, *SEM* standard error of the mean

### Sperm count and motility in the vas deferens and epididymis

The sperm count and sperm motility in both vas deferens and epididymis of all groups are presented in Table 2. The sperm count and motility were found to be decreased in both vas deferens and epididymis of the VC group (*P <* 0.05) compared with CTR group. Treatment with MAS 200 in VC rat attenuated the sperm count, sperm motility in both vas deferens and epididymis, significantly (*P <* 0.05).

**Table 2:** Effect of MAS 200 on sperm count, sperm motility in vas deference and epididymis in varicocele-induced male SD rats

	Mean sperm count (10^6^ /ml)		Sperm motility (%)	
	Vas deferens	Epididymis	Vas deferens	Epididymis
CTR (*n* = 10)	29.45 ± 2.14	43.35 ± 1.89	57.27 ± 4.19	36.12 ± 3.79
MAS 200 (*n* = 10)	23.70 ± 2.06	42.35 ± 2.21	55.63 ± 5.62	35.44 ± 3.55
VC (*n* = 10)	15.50 ± 1.72^***^	31.15 ± 3.08^**^	39.25 ± 3.35^**^	20.79 ± 2.95^*^
VC + MAS 200 (*n* = 10)	24.70 ± 2.63^##^	40.60 ± 1.93^#^	60.45 ± 3.47^##^	34.05 ± 2.59^#^

Data are presented in mean ± SEM. The differences were tested by one-way ANOVA followed by Tukey’s post hoc test; *n* = 10 for each group. ^*^Significant at *P* < 0.05; ^**^ Significant at *P* < 0.01; ^***^ Significant at *P* < 0.001- versus CTR group; ^#^ Significant at *P* < 0.05; ^##^Significant at *P* < 0.01; - versus VC group. *CTR* control, *MAS 200* MAS 200 mg/kg p.o., *VC* varicocele, *VC + MAS 200* MAS 200 mg/kg p.o., *p.o.* per oral, *ANOVA* analysis of variance, *SEM* standard error of the mean

### Histology of left testis and germ cell apoptosis

**Fig. 1:**
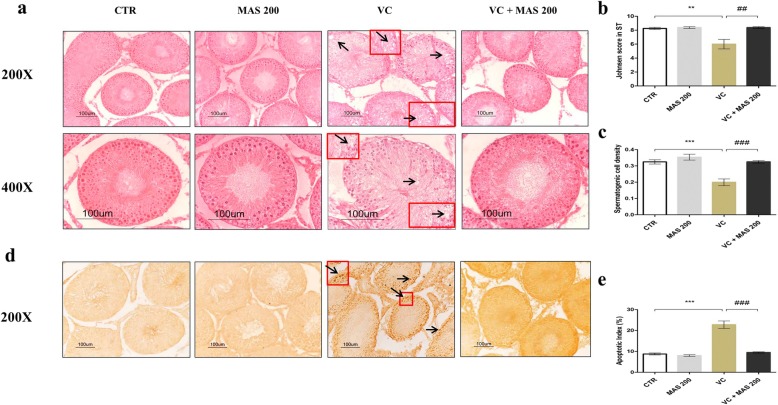
Effect of MAS on testicular histopathology and germ cell apoptosis based on with hematoxylin and eosin (H&E) staining, and terminal deoxynucleotidyl transferase-mediated dUTP nick-end labeling (TUNEL) staining of varicocele (VC)-induced male SD rats. **a** Hematoxylin and eosin stain showing scanty spermatogenic cells, irregular seminiferous tubules and seminiferous tubules with vacuoles in the VC group (arrowhead; **a**). **b** Johnsen’s score in seminiferous tubules compared among different groups. **c** Spermatogenic cell density of seminiferous tubules. (**d**) TUNEL staining. Black arrows showed TUNEL-positive cells (arrows; **d**). **e** Quantification of apoptotic index (AI) expressed as total positive cells/seminiferous tubule X 100. Data are presented as mean ± SEM. The differences were tested by one-way ANOVA followed by Tukey’s post hoc test; *n* = 10 for each group. ^*^Significant at *P* < 0.05; ^**^ Significant at *P* < 0.01; ^***^ Significant at *P* < 0.001- versus CTR group; ^#^ Significant at *P* < 0.05; ^##^ Significant at *P* < 0.01; ^###^ Significant at *P* < 0.001- versus VC group. CTR: control; MAS 200: MAS 200 mg/kg p.o.; VC: varicocele; VC + MAS 200: MAS 200 mg/kg p.o.; p.o.: per oral; ANOVA: analysis of variance; SEM: standard error of the mean; AI: apoptotic index

The H&E stating in testis from CTR and MAS 200 groups (Fig. 1a) showed no remarkable histopathological abnormalities and almost all seminiferous tubules showed complete spermatogenesis. The VC group showed few spermatogenic cells, irregular seminiferous tubules, vacuolization and fewer spermatozoa leaving a large cavity at the center of lumen. Treatment with MAS in the VC + MAS 200 group restored vacuolization, increased the germ cells and spermatozoa at the center of the lumen in seminiferous tubules. The Johnsen’s score declined in the VC group compared with CTR testis (*P <* 0.01). However, the Johnsen’s score in the testis of the VC group treated with MAS 200 increased higher than that of the VC group (*P <* 0.01) (Fig. 1b). Furthermore, the spermatogenic cell density was drastically reduced in the VC group compared with the CTR group (*P <* 0.001) (Fig. 1c). Treatment of VC rats with MAS 200 markedly increased the spermatogenic cell density compared with the VC rats (*P <* 0.001). The TUNEL assay results (Fig. 1d) showed that the apoptotic index (AI) was significantly upregulated in the VC group (*P <* 0.001) compared with the CTR group. MAS 200 treatment significantly reduced the VC-induced apoptosis in VC rats (*P <* 0.001).

### Lipid peroxidation, antioxidant enzymes and cytokine levels in left testis

The MDA, ROS/RNS levels, antioxidant activity of enzymes including SOD, GPx and catalase, and the levels of inflammatory cytokines IL-6 and TNF-α are presented in Table 3. As a lipid peroxidation marker, MDA levels and ROS/RNS levels were markedly elevated in the VC group (*P <* 0.05) compared with the CTR group. However, treatment of VC rat with MAS 200 significantly reduced MDA and ROS/RNS levels compared to the VC group. In addition, the enzymatic activities of SOD, GPx, as frontline antioxidant enzymes were downregulated in the VC group relative to the corresponding CTR group (*P <* 0.05). Another antioxidant enzyme catalase was also decreased compared with the CTR group. However, the data was not statistically significant with the CTR group. Antioxidant enzymes SOD, GPx and catalase were markedly increased in the VC + MAS 200 group compared with VC group (*P <* 0.05). To further investigate weather VC induced inflammation in testis tissue, the expression of TNF-α and IL-6 was analyzed. The expression of TNF-α and IL-6, a key inflammatory cytokine, was significantly elevated in the VC group compared with the CTR group (*P <* 0.01). Exposure of VC rats to MAS 200 markedly downregulated the inflammatory cytokines TNF-α and IL-6 (*P <* 0.01).

**Table 3:** Effect of MAS 200 on biomarkers of oxidative stress and inflammatory in varicocele-induced male SD rats

Parameter	CTR	MAS 200	VC	VC + MAS 200
MDA (μmole/mg protein)	7.37 ± 0.07	6.26 ± 0.69	11.38 ± 0.51^*^	5.46 ± 1.05^##^
ROS/RNS (nanomole DCF/ mg protein)	86.71 ± 10.91	83.55 ± 9.68	181.84 ± 10.51^***^	67.51 ± 11.88^###^
SOD level (units/mg protein)	8.32 ± 0.26	7.03 ± 0.63	4.12 ± 0.30^***^	6.10 ± 0.47^#^
GPx (nanomoles/min/mg protein)	33.44 ± 2.25	40.17 ± 3.02	23.82 ± 2.19^*^	37.72 ± 1.75^##^
Catalase (nanomoles/min/mg protein)	122.01 ± 8.75	138.32 ± 14.56	81.81 ± 12.82	149.03 ± 8.37^##^
IL-6 (pg/mg protein)	1636.12 ± 111.90	1779.21 ± 45.16	3165.71 ± 489.91^**^	1552.65 ± 153.45^##^
TNF-α (pg/mg protein)	962.54 ± 52.38	889.23 ± 42.60	1684.47 ± 94.90^***^	920.37 ± 37.06^###^

Data are presented in mean ± SEM. The differences were tested by one-way ANOVA followed by Tukey’s post hoc test; *n* = 10 for each group. ^*^Significant at *P* < 0.05; ^**^ Significant at *P* < 0.01; ^***^ Significant at *P* < 0.001- versus CTR group; ^#^Significant at *P* < 0.05; ^##^Significant at *P* < 0.01; ^###^Significant at *P* < 0.001- versus VC group. *CTR* control, *MAS 200* MAS 200 mg/kg p.o., *VC* varicocele, *VC + MAS 200* MAS 200 mg/kg p.o., *MDA* Malondialdehyde, *ROS/RNS* reactive oxygen species/reactive nitrogen species, *SOD* superoxide dismutase, *GPx* glutathione peroxidase, *IL-6* interleukin-6, *TNF-α* tumor necrosis factor- α, *p.o.* per oral, *ANOVA* analysis of variance, *SEM* standard error of the mean

### Hormone assays, hematology and serum biochemical markers

The serum testosterone, LH, FSH levels, hematology, and biochemical markers are presented in Table 4. The levels of serum testosterone in VC group were downregulated compared with both CTR and MAS 200 groups. However, the data were not statistically significant. Treatment with MAS 200 significantly improved the testosterone levels compared with the VC group (*P <* 0.05). A significant upregulation of serum LH and FSH levels was observed in the VC group compared with the CTR group (*P <* 0.001), which was downregulated by MAS 200 treatment of in VC rats (*P <* 0.01). The levels of white blood cells (WBC), red blood cells (RBC), hemoglobin (Hb), hematocrit (Hct), AST and ALT showed no significant effect at all treatment doses.

**Table 4:** Effect of MAS 200 on biomarkers of blood and serum hormone concentration in varicocele-induced male SD rats

Parameter	CTR	MAS 200	VC	VC + MAS 200
Serum testosterone (ng/ml)	1.76 ± 0.21	2.87 ± 1.04	1.05 ± 0.15	3.34 ± 0.45^#^
Serum LH (mIU/ml)	30.47 ± 2.63	22.97 ± 1.18	61.23 ± 3.83^***^	23.27 ± 1.33^###^
Serum FSH (ng/ml)	3.12 ± 0.26	4.15 ± 0.43	7.44 ± 0.39^***^	4.81 ± 0.35^##^
WBC (× 10^3^/μL)	8.18 ± 0.32	7.70 ± 0.36	8.10 ± 0.44	7.61 ± 0.53
RBC (× 10^4^/μL)	7.92 ± 0.13	7.94 ± 0.10	7.83 ± 0.07	7.72 ± 0.14
Hb (g/dL)	14.13 ± 0.16	13.57 ± 0.42	13.96 ± 0.40	13.73 ± 0.48
Hct (%)	43.60 ± 0.46	41.83 ± 0.43	43.19 ± 0.47	42.94 ± 0.44
AST (IU/L)	118.70 ± 13.53	128.80 ± 13.88	129.00 ± 8.05	115.40 ± 9.34
ALT (IU/L)	54.10 ± 6.51	56.20 ± 3.41	45.40 ± 2.49	45.60 ± 1.93

Data are presented in mean ± SEM. The differences were tested by one-way ANOVA followed by Tukey’s post hoc test; *n* = 10 for each group. ^*^Significant at *P* < 0.05; ^**^ Significant at *P* < 0.01; ^***^ Significant at *P* < 0.001- versus CTR group; ^#^Significant at *P* < 0.05; ^##^Significant at *P* < 0.01; ^###^Significant at *P* < 0.001- versus VC group. *CTR* control, *MAS 200* MAS 200 mg/kg p.o., *VC* varicocele, *VC + MAS 200* MAS 200 mg/kg p.o., *LH* luteinizing hormone, *FSH* follicle stimulating hormone, *WBC* white blood cell, *RBC* red blood cell, *Hb* hemoglobin, *Hct* hematocrit, *AST* aspartate aminotransferase, *ALT* alanine aminotransferase, *p.o.* per oral, *ANOVA* analysis of variance, *SEM* standard error of the mean

### Western blot and immunohistochemistry of proteins expressed in left testis

**Fig. 2:**
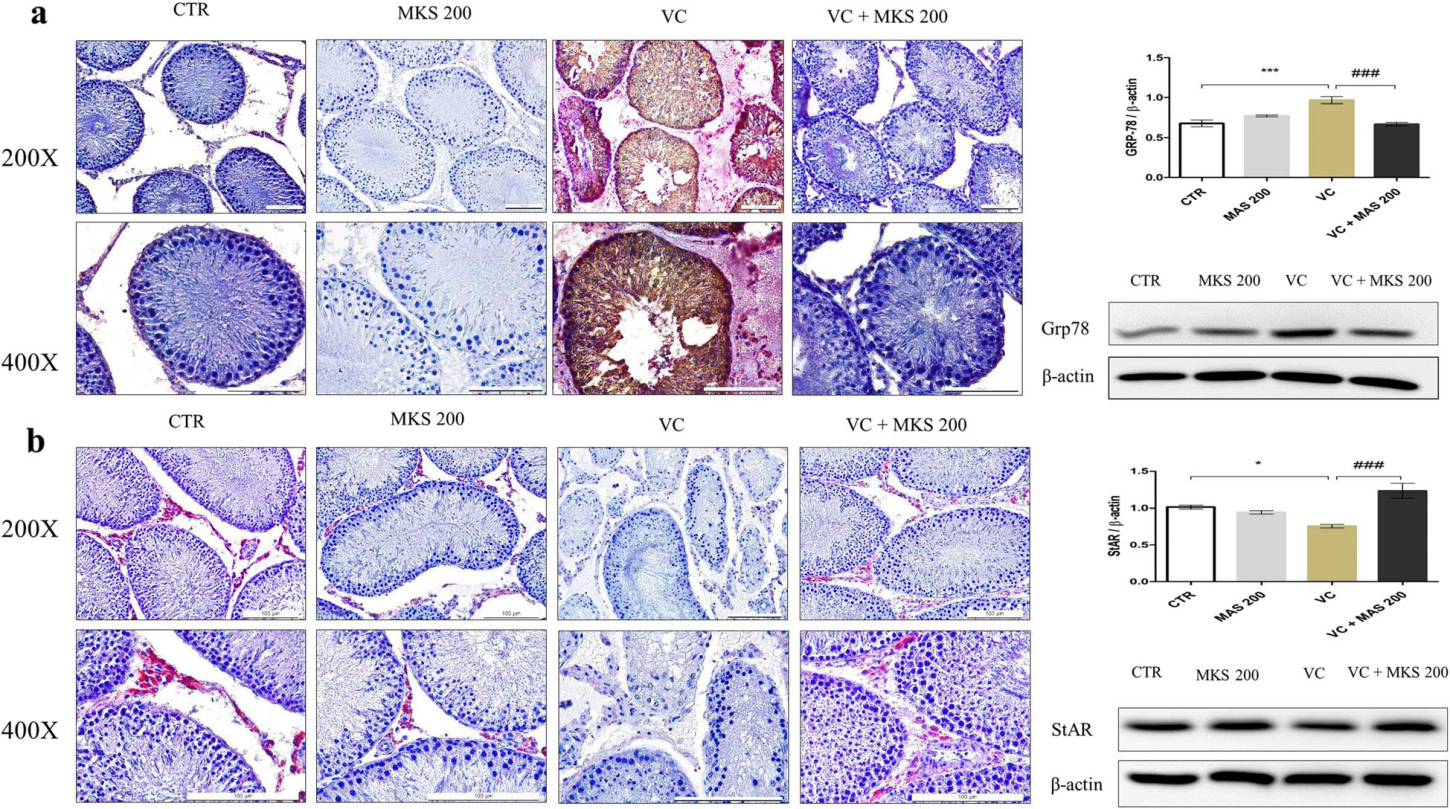
Effect of MAS on testicular protein levels in varicocele (VC)-induced male SD rats determined by immunohistochemistry and western blot analysis. (**a**) Grp 78, (**b**) StAR. Data are presented as means ± SEM. The differences were tested by one-way ANOVA followed by Tukey’s post hoc test; *n* = 10 for each group. ^*^Significant at *P* < 0.05; ^**^ Significant at *P* < 0.01; ^***^ Significant at *P* < 0.001- versus CTR group; ^#^ Significant at *P* < 0.05; ^##^ Significant at *P* < 0.01; ^###^ Significant at *P* < 0.001- versus VC group. CTR: control; MAS 200: MAS 200 mg/kg p.o.; VC: varicocele; VC + MAS 200: MAS 200 mg/kg p.o.; p.o.: per oral; ANOVA: analysis of variance; SEM: standard error of the mean

**Fig. 3:**
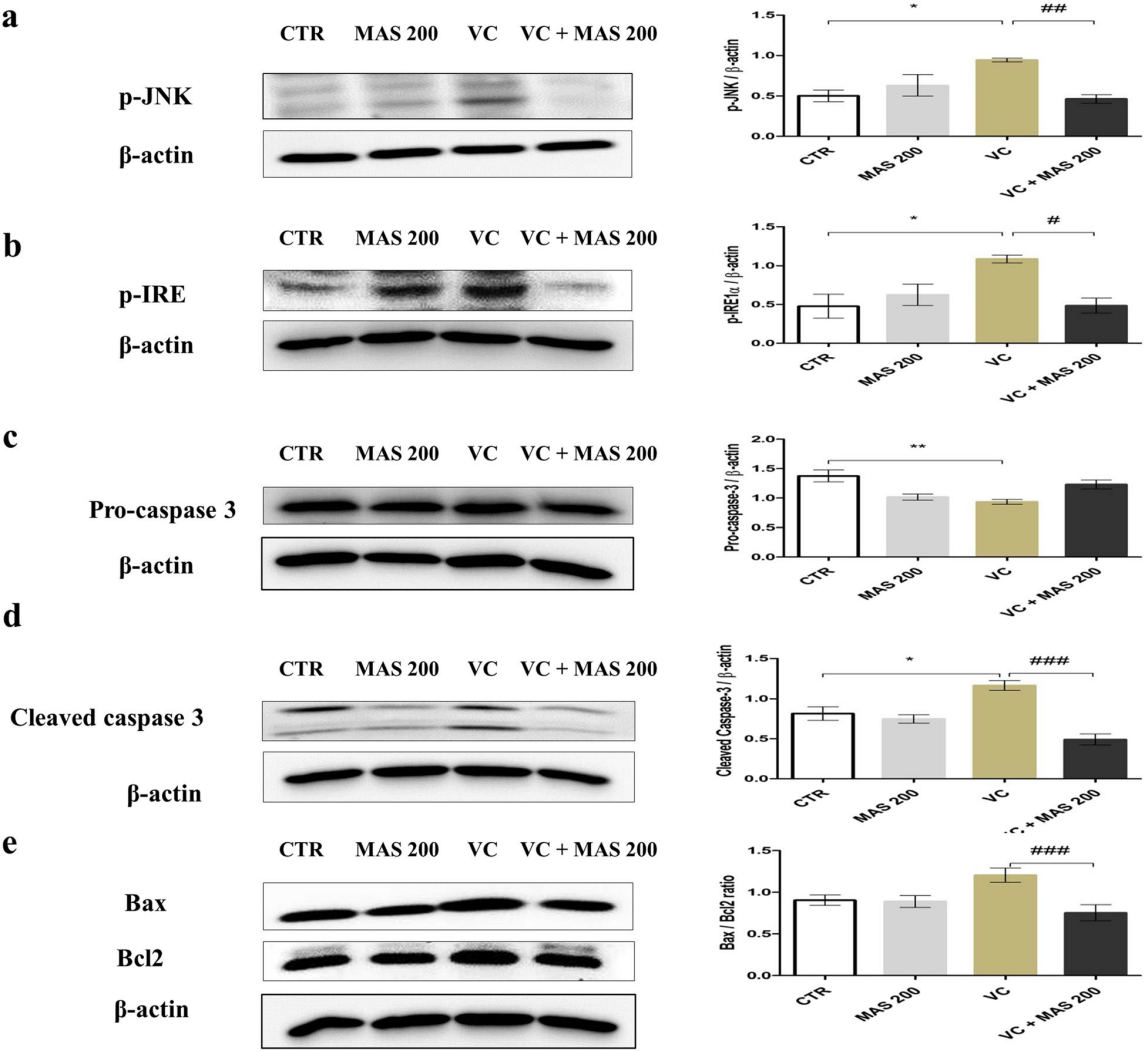
Effect of MAS on testicular protein levels in varicocele (VC)-induced male SD rats determined by western blot analysis. **a** p-JNK, (**b**) p-IRE, (**c**) Pro-caspase-3, (**d**) cleaved caspase-3, (**e**) Bax:Bcl 2 ratio. Data are presented in mean ± SEM. The differences were tested by one-way ANOVA followed by Tukey’s post hoc test; *n* = 10 for each group. ^*^Significant at *P* < 0.05; ^**^ Significant at *P* < 0.01; ^***^ Significant at *P* < 0.001- versus CTR group; ^#^ Significant at *P* < 0.05; ^##^ Significant at *P* < 0.01; ^###^ Significant at *P* < 0.001- versus VC group. CTR: control; MAS 200: MAS 200 mg/kg p.o.; VC: varicocele; VC + MAS 200: MAS 200 mg/kg p.o.; p.o.: per oral; ANOVA: analysis of variance; SEM: standard error of the mean

To analyze the protective mechanism of MAS against VC-induced testicular dysfunction, we investigated the protein expression of the ER stress markers Grp 78, p-JNK, p-IRE1 and apoptosis markers caspase 3, cleaved caspase-3, Bax:Bcl2 ratio by western blotting (Figs. 2 and 3). We also determined the expression of StAR, a testosterone biosynthesis marker, by western blotting (Fig. 2). The expression of Grp 78 and StAR protein was further confirmed by immunohistochemistry analysis (Fig. 2). In the CTR group, a low and basal activation of ER stress and apoptosis-related proteins was detected. In the VC group, the expression of Grp78, p-JNK, p-IRE and cleaved caspase-3 was significantly upregulated compared with the CTR group (Fig. 2a, Fig. 3a, b, and d) (*P <* 0.05). The level of pro caspase 3 was significantly decreased in VC group compared with the CTR group (Fig. 3c) (*P <* 0.01). The level of Bax:Bcl 2 ratio in VC group was upregulated compared with the CTR group (Fig. 3e). However, the data were statistically significant. In the presence of MAS 200, the activation of Grp 78, p-IRE1, P-JNK, cleaved caspase-3 and Bax: Bcl2 ratio was reversed (*P* < 0.05). The expression of StAR protein was downregulated in the VC group compared with the CTR group (Fig. 2b) (*P <* 0.05). However, the VC + MAS 200 group showed a significant improvement in StAR protein expression compared with VC group (*P <* 0.001). Moreover, immunostaining of Grp 78 was prominent in the VC group seminiferous tubules (Fig. 2a). MAS treatment reduced the expression of Grp 78 in VC rats, suggesting that MAS regulated ER stress pathway in VC rats. The expression of StAR protein was diminished in Leydig cells in the VC group compared with the CTR group (Fig. 2b). However, treatment with MAS 200 strongly induced StAR protein in the Leydig cells of VC rats.

## Discussion

Varicocele-induced testicular dysfunction and apoptosis is one of the main pathophysiological mechanisms underlying male infertility [29]. Rat models of experimental varicocele showed germ cell apoptosis in the testis, triggered by oxidative stress, and increased hypoxia-inducible factor-1α, which altered testicular histology and caused sperm DNA fragmentation [16, 29]. Men with varicocele are at high risk of developing infertility due to testicular dysfunction. Therefore, strategies that effectively preserve fertility during varicocele or post varicocelectomy are needed. Antioxidant therapy may be considered as the treatment modality for varicocele, however, none of them showed promising results in humans [17, 22, 30, 31]. In the present study, we investigated the crosstalk between ER stress and mitochondrial apoptosis in VC-induced testicular dysfunction and determined the efficacy of MAS to ameliorate infertility.

The results indicated that a VC-induced SD rat showed no difference in body or reproductive organ weights except testicular weight. Testicular weight in VC rats was decreased compared with the CTR group, consistent with previous findings [32]. The VC group showed a decline in sperm count and motility as well as morphological aberration in seminiferous tubules. Johnsen’s score and spermatogenic cell density were downregulated in the experimental VC group. These results were consistent with our previous findings and reported literature [8, 32, 33]. MAS improved sperm count and motility, testicular morphology, such as Johnsen’s score and spermatogenic cell density and may represent a promising therapeutic option to improve infertility. Results of RBC count, WBC count, Hb, Hct, AST and ALT showed no difference between the different groups, consistent with previous findings [8].

Testosterone is synthesized in Leydig cells, which play an essential role in spermatogenesis [33]. Testicular dysfunction due to VC compromises normal endocrine function of Leydig cells. StAR plays an indispensable role as a rate-limiting step of testosterone biosynthesis [11]. StAR mediates the cholesterol transport across the mitochondrial membrane and conversion into pregnenolone in the inner membrane of mitochondria by cytochrome p450 side chain cleavage enzyme, which is further converted by series of enzymes to testosterone and other steroid hormones [33]. In the present study, the serum testosterone levels were downregulated in the VC group. Furthermore, the downregulation of StAR protein observed in western blots and immunohistochemistry of the VC group established the suppression of testosterone biosynthesis. LH and FSH are the main regulatory hormones for testosterone stimulation in males [34]. The serum levels of LH and FSH were elevated in the VC group, probably due to a feedback mechanism of hypothalamus-pituitary-testicular axis regulating steroidogenesis and spermatogenesis [33]. MAS upregulated the serum testosterone and StAR protein expression in VC rats, and decreased serum LH and FSH levels, suggesting that MAS exhibits androgenic activity.

Oxidative stress is a widely accepted mechanism of testicular germ cell apoptosis linking varicocele with male infertility [35]. In our study, oxidative stress markers MDA and ROS/RNS were upregulated in testicular tissue of VC rats. The present findings are similar with previous report [8]. Increased ROS is associated with decreased sperm count, motility, altered morphology and sperm DNA fragmentation [36, 37]. Moreover, antioxidant enzyme activities of SOD, GPX and catalase levels were also downregulated in the VC group. Our results are consistent with available evidence [17]. Treatment with MAS 200 reversed all the biochemical parameters in VC rats, suggesting that MAS scavenged free radicals and ameliorated lipid peroxidation in VC rats. Furthermore, AI value of germ cell apoptosis was determined by TUNEL assay and in VC group, AI was higher than in the CTR group. Our recent study showed that VC induced ROS-mediated ER stress in testis [8]. Increase ROS play a bidirectional role in oxidative stress and ER stress and result in unfolded protein accumulation in the ER [38]. ER stress triggers apoptosis via three signaling pathways: IRE1, ATF 6 and PERK [39]. In the present study, we investigated IRE1 pathway based on a previous report of IRE1-p-JNK pathway-mediated testicular apoptosis in VC-induced rat [8]. Prolonged ER stress was associated with apoptosis and mediated primarily via PERK and IRE1 signaling pathway [15]. Furthermore, ER stress chaperone GRP 78 predominantly occurs in pachytene spermatocytes, suggesting that ER stress signaling plays an indispensable role in spermatogenesis [40]. Several studies reported that ER stress was associated with male reproductive dysfunction [41–43]. Our study showed that VC induced upregulation of IRE1 pathway molecules grp 78, p-JNK and p-IRE 1, which was reduced by the treatment of MAS 200 in VC rats. The results of our study are similar to our previous findings [8]. Furthermore, we investigated the crosstalk between ER stress and mitochondrial apoptosis by analyzing caspase activity and Bax:Bcl2 expression. The upregulation of cleaved caspase-3 and Bax:Bcl2 ratio in the VC group suggests germ cell apoptosis via mitochondrial apoptosis [16]. Acceleration of Bcl2 in germ cell favors cell survival, whereas elevation of Bax accelerates cell death [17]. Activation of JNK promotes translocation of Bax from cytosol to mitochondria and plays an important role in the release of cytochrome c from mitochondrial inner membrane into the cytosol and subsequent apoptosis [44]. The study results are consistent with previous findings [16, 17, 28]. Treatment with MAS 200 ameliorates AI of germ cell via downregulation of cleaved caspase-3 and Bax:Bcl2 ratio, suggest that MAS has a protective role against mitochondrial apoptosis.

The current study relates to the lack of assessment of varicocele effect in both testicles. We did not investigate the bilateral testicular effects in the present study. We considered this parameter as an experimental variable. Furthermore, the levels of pro-inflammatory cytokines IL-6 and TNF-α in the left testis were higher in VC rats than in CTR rats. Testicular inflammation might be attributed to testicular hypoxia and increased testicular temperature in rats with varicocele [22, 45]. However, MAS treatment downregulated the inflammatory markers, which suggested that MAS exerted anti-inflammatory activities.

## Conclusion

**Fig. 4:**
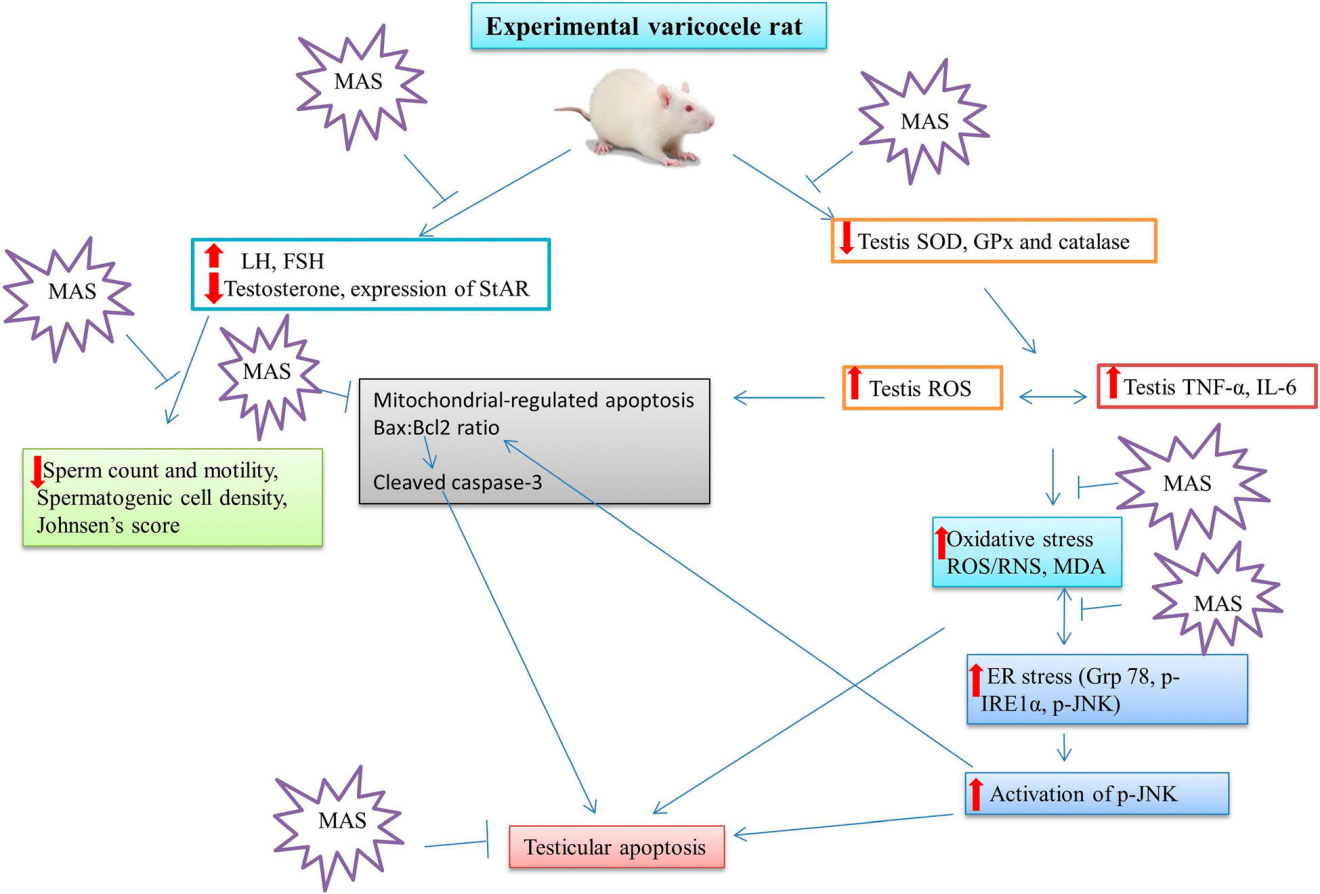
Schematic diagram showing molecular mechanism of varicocele-induced testicular dysfunction and its prevention by MAS. ER: Endoplasmic reticulum; ROS/RNS: Reactive oxygen species/reactive nitrogen species; MDA: Malondialdehyde; SOD: Superoxide dismutase; GPx: Glutathione peroxidase; IL-6: Interleukin-6; TNF-α: Tumor necrosis factor-α; GRP-78: Glucose-regulated protein-78; p-JNK: Phosphorylated c-Jun-N-terminal kinase; p-IRE1α: Phosphorylated Inositol-Requiring Transmembrane Kinase/Endoribonuclease 1α; JNK: C-jun-N-terminal kinase; Bax: BCL 2 associated X protein; Bcl-2: B-cell lymphoma 2**;** StAR: Steroidogenic acute regulatory protein

The present findings suggest a crosstalk between oxidative stress, ER stress and mitochondrial pathway in varicocele-induced testicular germ cell apoptosis (Fig. 4). MAS promotes spermatogenesis in varicocele-induced SD rat, probably by decreasing cytokine (interleukin- 6, and TNF-α) levels, regulating abnormal sex hormones, and decreasing oxidative stress, ER stress and apoptosis. Our findings provide new insights into the ameliorative effects of MAS in testicular dysfunction associated with varicocele. MAS may represent a complementary medicine for the treatment of varicocele and male infertility.

## Supplementary information

**Additional file 1: Figure S1.** HPLC chromatograms and ESI-MS spectra of MOTILIPERM and different herbal ingredients. Peaks: monotropein (1), deacetylasperulosidic acid (2), quercetin 3,4′-diglucoside (3), hyperoside (4), astragalin (5), spiraeoside (6) and quercetin (7). HPLC: high performance liquid chromatography; ESI-MS: electrospray ionization mass spectrometry.

**Additional file 2: Figure S2.** Identification of major compounds in MOTILIPERM by ESI-MS spectral data. (A) monotropein (**1**) and deacetylasperulosidic acid (**2**) from *Morinda officinalis* (B) quercetin 3,4'′-diglucoside (**3**) spiraeoside (**6**) and quercetin (**7**) from *Allium cepa* (C) hyperoside (**4**) and astragalin (**5**) from *Cuscuta chinensis*. ESI-MS: electrospray ionization mass spectrometry

**Additional file 3: Table S1.** Linear regression data, LOD and LOQ of investigated components 1, **5** and **6** in MOTILIPERM.

**Additional file 4: Table S2.** Intra- and inter-day variability for the assay of three investigated components in MOTILIPERM.

**Additional file 5: Table S3.** Contents (mg/g) of three compounds **1**, **5** and **6** in MOTILIPERM and each herb.

## Data Availability

All the data is contained in the manuscript, raw datasets used and/or analyzed during the current study is available from the corresponding author on reasonable request.

## Abbreviations

ALT: Alanine aminotransferase
ANOVA: Analysis of variance
AST: Aspartate aminotransferase
Bax: BCL 2 associated X protein
Bcl-2: B-cell lymphoma 2
CTR: Control
ER: Endoplasmic reticulum
FSH: Follicle stimulating hormone
GPx: Glutathione peroxidase
GRP-78: Glucose-regulated protein-78
Hb: Hemoglobin
Hct: Hematocrit
IL-6: Interleukin-6
JNK: C-jun-N-terminal kinase
LH: Luteinizing hormone
MAS 200: MAS 200 mg/kg p.o.
MAS: Monotropein, astragalin, and spiraeoside
MDA: Malondialdehyde
p.o.: Per oral
p-IRE1α: Phosphorylated inositol-requiring transmembrane kinase/endoribonuclease 1α
p-JNK: Phosphorylated c-Jun-N-terminal kinase
RBC: Red blood cell
ROS/RNS: Reactive oxygen species/reactive nitrogen species
SEM: Standard error of the mean
SOD: Superoxide dismutase
StAR: Steroidogenic acute regulatory protein
TNF-α: Tumor necrosis factor- α
VC + MAS 200: MAS 200 mg/kg p.o.
VC: Varicocele
WBC: White blood cell
