# Supplementary figures and images for: The ameliorative effect of monotropein, astragalin, and spiraeoside on oxidative stress, endoplasmic reticulum stress, and mitochondrial signaling pathway in varicocelized rats
Source: BMC Complement Altern Med. 2019 Nov 26;19:333. doi: 10.1186/s12906-019-2736-9 (PMC6880392; doi:10.1186/s12906-019-2736-9)

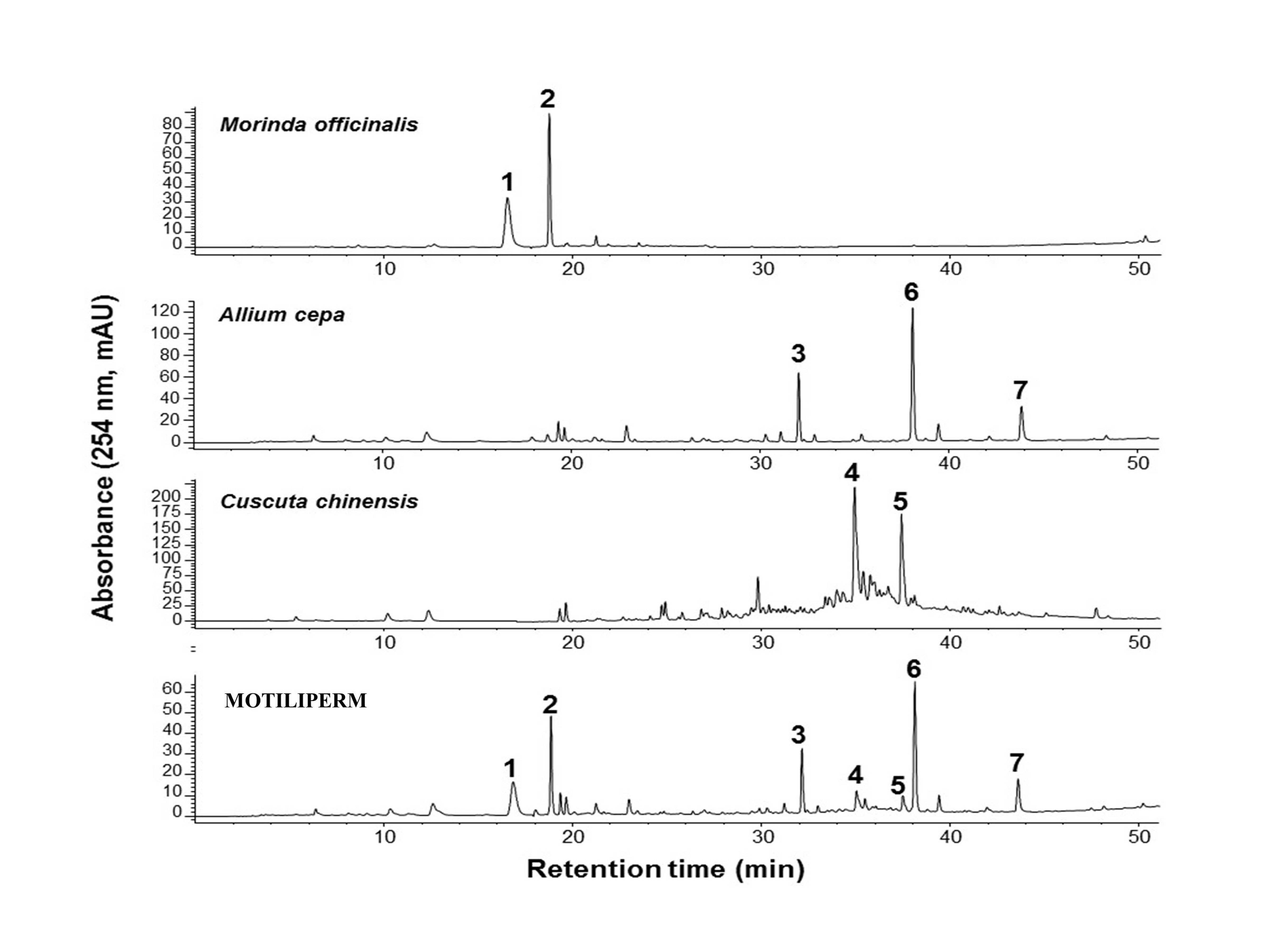

Supplement: Supplementary file 1 — Additional file 1: Figure S1. HPLC chromatograms and ESI-MS spectra of MOTILIPERM and different herbal ingredients. Peaks: monotropein (1), deacetylasperulosidic acid (2), quercetin 3,4′-diglucoside (3), hyperoside (4), astragalin (5), spiraeoside (6) and quercetin (7). HPLC: high performance liquid chromatography; ESI-MS: electrospray ionization mass spectrometry. [file 12906_2019_2736_MOESM1_ESM.jpg]

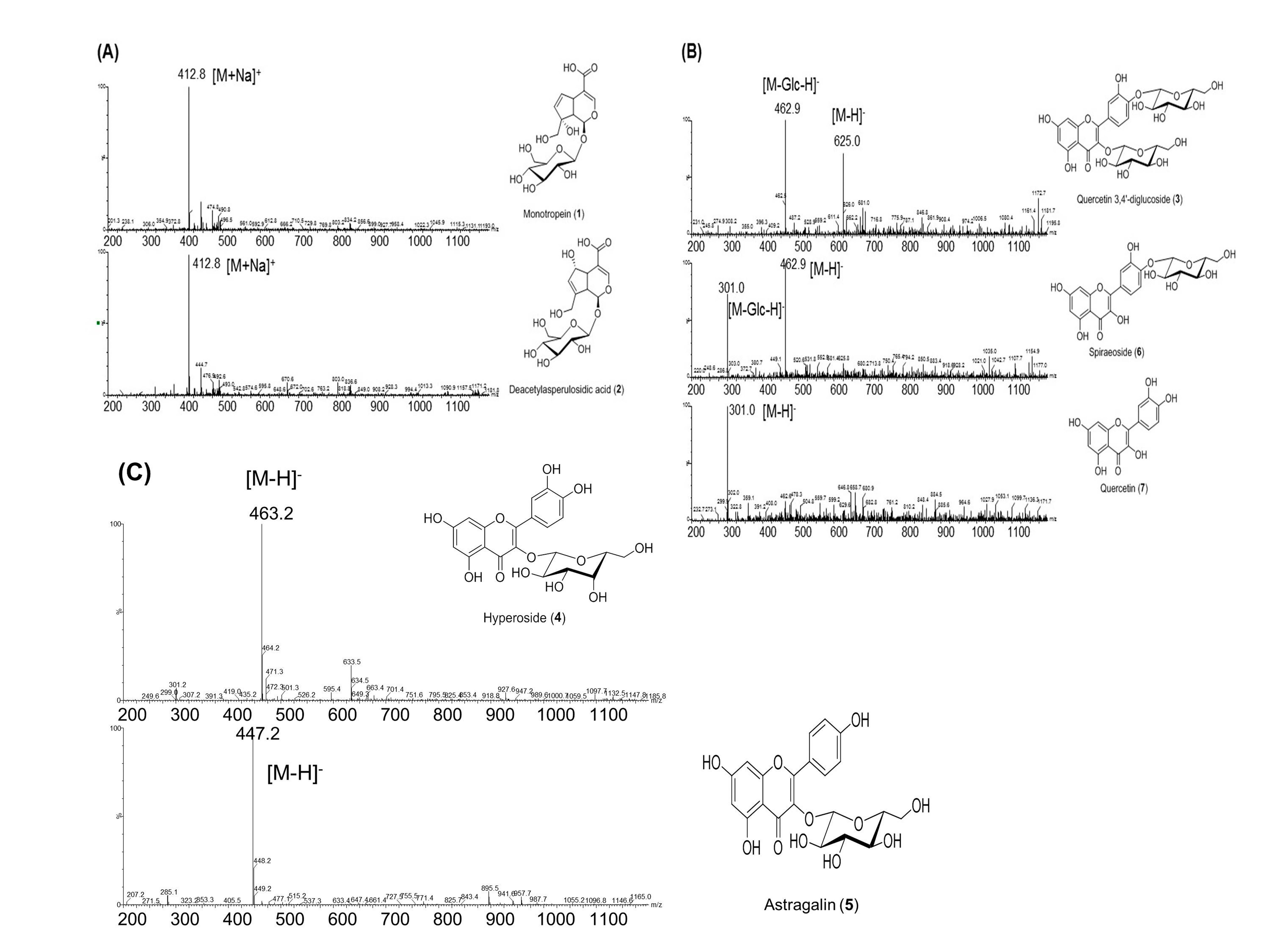

Supplement: Supplementary file 2 — Additional file 2: Figure S2. Identification of major compounds in MOTILIPERM by ESI-MS spectral data. (A) monotropein (1) and deacetylasperulosidic acid (2) from Morinda officinalis (B) quercetin 3,4'′-diglucoside (3) spiraeoside (6) and quercetin (7) from Allium cepa (C) hyperoside (4) and astragalin (5) from Cuscuta chinensis. ESI-MS: electrospray ionization mass spectrometry [file 12906_2019_2736_MOESM2_ESM.jpg]
