# Supplementary material for: The ameliorative effect of monotropein, astragalin, and spiraeoside on oxidative stress, endoplasmic reticulum stress, and mitochondrial signaling pathway in varicocelized rats
Source: BMC Complement Altern Med. 2019 Nov 26;19:333. doi: 10.1186/s12906-019-2736-9 (PMC6880392; doi:10.1186/s12906-019-2736-9)
Supplement: Supplementary file 3 — Additional file 3: Table S1. Linear regression data, LOD and LOQ of investigated components 1, 5 and 6 in MOTILIPERM. [file 12906_2019_2736_MOESM3_ESM.docx]

**Table S1**. Linear regression data, LOD and LOQ of investigated components 1, **5** and **6** in MOTILIPERM

| **Analytes** | **Linear regression data** | | | **LOD** | **LOQ** |
| --- | --- | --- | --- | --- | --- |
|  | **Regressive equation** | **Test range**  **(mg/ml)** | **r^2^** |  |  |
| **Monotropein (1)** | y = 6,158,713 x +16,818 | 0.025 – 0.4 | 0.9998 | 0.0051 | 0.0153 |
| **Astragalin (5)** | y = 17,364,521 x + 790 | 0.00175 – 0.028 | 0.9999 | 0.0002 | 0.0006 |
| **Spiraeoside (6)** | y = 15,438,428 x + 1,373 | 0.02 - 0.32 | 1.0000 | 0.0007 | 0.0022 |

LOC: limit of detection; LOQ: limit of quantification.
