# Supplementary material for: The ameliorative effect of monotropein, astragalin, and spiraeoside on oxidative stress, endoplasmic reticulum stress, and mitochondrial signaling pathway in varicocelized rats
Source: BMC Complement Altern Med. 2019 Nov 26;19:333. doi: 10.1186/s12906-019-2736-9 (PMC6880392; doi:10.1186/s12906-019-2736-9)
Supplement: Supplementary file 4 — Additional file 4: Table S2. Intra- and inter-day variability for the assay of three investigated components in MOTILIPERM. [file 12906_2019_2736_MOESM4_ESM.docx]

**Table S2**. Intra- and inter-day variability for the assay of three investigated components in MOTILIPERM

| **Analytes** | **Concentration**  **(mg/ml)** | **Intra-day( *n*= 5)** | | | **Inter-day(*n* = 5)** | | |
| --- | --- | --- | --- | --- | --- | --- | --- |
|  |  | **Observed concentration (*n = 5*)**  **Mean ± SD** | **Accuracy (%)** | **Precision (%)** | **Observed concentration (*n = 5*)**  **Mean ± SD** | **Accuracy (%)** | **Precision (%)** |
| **Monotropein (1)** | 25 | 23.61 ± 0.28 | 94.46 | 1.20 | 23.68 ± 1.32 | 94.72 | 5.59 |
|  | 100 | 101.23 ± 1.11 | 101.24 | 1.10 | 102.72 ± 2.70 | 102.72 | 2.63 |
|  | 400 | 411.47 ± 4.36 | 102.87 | 1.06 | 405.83 ± 6.28 | 101.46 | 1.55 |
| **Astragalin (5)** | 1.75 | 1.74 ± 0.02 | 99.60 | 1.07 | 1.76 ± 0.03 | 100.64 | 1.48 |
|  | 7 | 6.98 ± 0.05 | 99.72 | 0.73 | 7.22 ± 0.33 | 103.17 | 4.63 |
|  | 28 | 27.94 ± 0.21 | 99.80 | 0.76 | 27.86 ± 0.52 | 99.51 | 1.87 |
| **Spiraeoside (6)** | 20 | 20.33 ± 0.11 | 101.66 | 0.52 | 20.02 ± 0.39 | 100.15 | 1.93 |
|  | 80 | 80.27 ± 0.33 | 100.35 | 0.41 | 80.53 ± 1.70 | 100.67 | 2.11 |
|  | 320 | 321.97 ± 1.79 | 100.62 | 0.55 | 321.63 ± 0.44 | 100.51 | 0.14 |
