# Supplementary material for: The ameliorative effect of monotropein, astragalin, and spiraeoside on oxidative stress, endoplasmic reticulum stress, and mitochondrial signaling pathway in varicocelized rats
Source: BMC Complement Altern Med. 2019 Nov 26;19:333. doi: 10.1186/s12906-019-2736-9 (PMC6880392; doi:10.1186/s12906-019-2736-9)
Supplement: Supplementary file 5 — Additional file 5: Table S3. Contents (mg/g) of three compounds 1, 5 and 6 in MOTILIPERM and each herb. [file 12906_2019_2736_MOESM5_ESM.docx]

**Table S3.** Contents (mg/g) of three compounds **1**, **5** and **6** in MOTILIPERM and each herb

| **Extract (mg/g)** | **Monotropein (1)** | **Astragalin (5)** | **Spiraeoside (6)** |
| --- | --- | --- | --- |
| *Morinda officinalis* | 11.45 ± 0.07 |  |  |
| *Allium cepa* |  |  | 7.44 ± 0.05 |
| *Cuscuta chinensis* |  | 8.49 ± 0.09 |  |
| MOTILIPERM | 6.69 ± 0.19 | 0.41 ± 0.02 | 3.61 ± 0.08 |
